# Effectiveness of a Health Education Program for Patients Who Had a Stroke and Their Caregivers by Controlling Modifiable Risk Factors to Reduce Stroke Recurrence in a Tertiary Hospital in Bangladesh: Protocol for a Randomized Controlled Trial

**DOI:** 10.2196/51178

**Published:** 2023-12-15

**Authors:** Mahabuba Afrin, Sharif Uddin Khan, Subir Chandra Das, K A T M Ehsanul Huq, Michiko Moriyama

**Affiliations:** 1 Department of Health Science Graduate School of Biomedical and Health Sciences Hiroshima University Hiroshima Japan; 2 Department of Neurology National Institute of Neurosciences & Hospital Dhaka Bangladesh

**Keywords:** stroke, caregiver, recurrence of stroke, health education, Bangladesh, modifiable risk factor, recurrence, hospital, disability, lifestyle change, behavioral change, risk factor, quality of life, tertiary

## Abstract

**Background:**

Stroke is a significant public health concern that causes severe and long-lasting disability. The recurrence of stroke is increasing due to lack of patients’ knowledge and compliance with treatment to control modifiable risk factors and lifestyle changes. Health education can be an effective way to increase knowledge about behavioral changes in patients who had a stroke.

**Objective:**

The aim of this study is to evaluate the effectiveness of a health education program in Bangladesh for patients who had their first stroke and their family caregivers in order to reduce the recurrence of stroke by controlling modifiable risk factors.

**Methods:**

This is a parallel, open-label, prospective randomized controlled trial conducted at the National Institute of Neurosciences & Hospital in Dhaka, Bangladesh. We enrolled ≥18-year-old patients of both sexes who had a history of first stroke attack (transient ischemic attack, hemorrhagic stroke, lacunar stroke, atherothrombotic stroke, or cardioembolic stroke). We stratified patients into those aged <65 years and those aged ≥65 years and according to types of strokes for randomization. Our estimated sample size was 432. The intervention group receives face-to-face counseling on lifestyle modifications, blood pressure monitoring, and medication compliance–related health education during enrollment and monthly follow-ups. However, the control group receives only usual care from the hospital. The primary end point of this study is the reduction in the stroke recurrence rates at the end of the 12-month follow-up period. The secondary end points are to (1) assess the occurrence of all adverse events such as other cardiac events and all-cause mortality, (2) evaluate the status of the controlling modifiable risk factors such as blood pressure, glycated hemoglobin, and non–high-density lipoprotein cholesterol, (3) assess the knowledge related to stroke by using the investigator-developed questionnaire, (4) evaluate the lifestyle behavior related to stroke by using the investigator-developed questionnaire, (5) assess medication adherence, and (6) evaluate the quality of life of patients who had a stroke and their family caregivers. The evaluation points will be at baseline, 6 months, and 12 months for both groups.

**Results:**

Patients’ enrollment started on October 2022, and follow-up will be completed in March 2024. A total of 432 participants were included in both the intervention (n=216) and control groups (n=216). This study was approved by the institutional review board and the ethics review board of the National Institute of Neurosciences & Hospital (IRB/NINSH/2022/151) on August 30, 2022.

**Conclusions:**

Our health education program is expected to reduce the recurrence of stroke and improve the quality of life of patients who have had the first stroke. The results of this study will provide insights into the importance of health education for (self)-management and prevention of stroke.

**Trial Registration:**

ClinicalTrials.gov NCT05520034; https://clinicaltrials.gov/ct2/show/NCT05520034

**International Registered Report Identifier (IRRID):**

DERR1-10.2196/51178

## Introduction

### Background

Stroke is a major global health concern, ranked as the second highest cause of death in 2019 [1], with an estimate of 93.2-110.5 million people affected worldwide [2]. This debilitating condition causes long-term physical, mental, and social disability, leading to financial difficulties for patients and their families [2,3]. People are affected by stroke in both low-income and high-income countries, and most cases have been reported in older age groups [4,5], although it has also been reported in younger individuals, especially in low-income countries [6]. In 2019, the burden of stroke increased from 70% to 85% between 1990 and 2019, and the mortality rates rose by 43% during this period. Among them, 62.4% experienced an ischemic stroke, 27.9% had an intracerebral hemorrhage, and 9.7% had subarachnoid hemorrhage, with corresponding mortality rates of 4%, 0.36%, and 0.36%, respectively, resulting in a total death rate of 6% [2]. A meta-analysis of 26 studies from 1997 to 2019 found stroke recurrence rates ranging from 5.7% to 51.3% over 1-10 years, with a 1-year recurrence rate of 5.7%-17.7% [7]. The first 3 months following the initial attack are the most critical for recurrence [8]. Moreover, the meta-analysis indicated that factors such as diabetes mellitus, smoking status, peripheral artery disease, hypercoagulable state, depression, 24-hour minimum systolic blood pressure (BP), 24-hour maximum diastolic BP, age, family history of stroke, National Institutes of Health Stroke Scale score status, hypertension, and history of cardiac disease were found to influence the recurrence of stroke [9,10].

In Bangladesh, stroke is the topmost cause of death, accounting for 82.31 deaths per 100,000 people in 2019 across all age groups and genders [11], with prevalence of approximately 11.39 cases per 1000 people [4]. Among them, 79.7% experienced ischemic stroke, 15.7% had hemorrhagic stroke, and 4.6% were diagnosed with subarachnoid hemorrhage [4]. A hospital-based study in Bangladesh reported cumulative recurrence rates of 14.7% at 3 months, 15.3% at 6 months, 17.3% at 9 months, and 20% at 1 year, predominantly in individuals older than 75 years [8]. Male patients with hypertension, diabetes, and dyslipidemia had a higher risk of recurrence compared to females. Additional risk factors included hypertension (79.2%), dyslipidemia (38.9%), tobacco use and smoking (37.2%), diabetes (28.8%), ischemic heart disease (20.1%), atrial fibrillation (13.9%), and obesity (9.7%) [4].

Secondary preventive measures must be implemented that address comorbidities such as hypertension and diabetes through lifestyle modifications, surgical interventions, and pharmacological interventions to reduce the recurrence of stroke [12]. Health education interventions can effectively enhance knowledge, raise awareness, modify lifestyle behaviors, and control modifiable risk factors, ultimately improving the quality of life (QoL). A study demonstrated that a 6-month health education program reduced stroke recurrence rate by 50% at 30-month follow-up [13]. Furthermore, regular postdischarge checkups and early follow-ups are necessary to prevent complications and further recurrence. Many countries worldwide have implemented stroke prevention efforts [14]. A randomized controlled study showed that both face-to-face and telehealth education programs resulted in significant improvements in health behaviors related to physical activity, nutrition, low-salt diets, and medication adherence. Additionally, the intervention group exhibited decreased systolic BP and cholesterol levels [15,16]. Another similar study using a randomized controlled design found that a web-based stroke education program led to significant increases in exercise, reduced consumption of salty foods, increased intake of fruits and vegetables, enhanced sense of control, improved health motivation, and improved caregiver mastery. However, all these interventions did not result in significant reduction of stroke recurrence [17]. In Bangladesh, a significant challenge for patients who have had a stroke is the absence of a follow-up system after hospital discharge and lack of patient and family education during hospital stay or in postdischarge or primary care settings. As a result, low medication compliance and poor risk factor control have been reported among patients who have had a stroke in Bangladesh [18].

### Study Objectives

The objective of this study is to evaluate the effectiveness of a health education program for patients who had their first stroke (transient ischemic attack, hemorrhagic stroke, lacunar stroke, atherothrombotic stroke, or cardioembolic stroke) and their family caregivers in order to reduce the recurrence of stroke.

### Study Framework and Hypothesis

**Figure 1:**
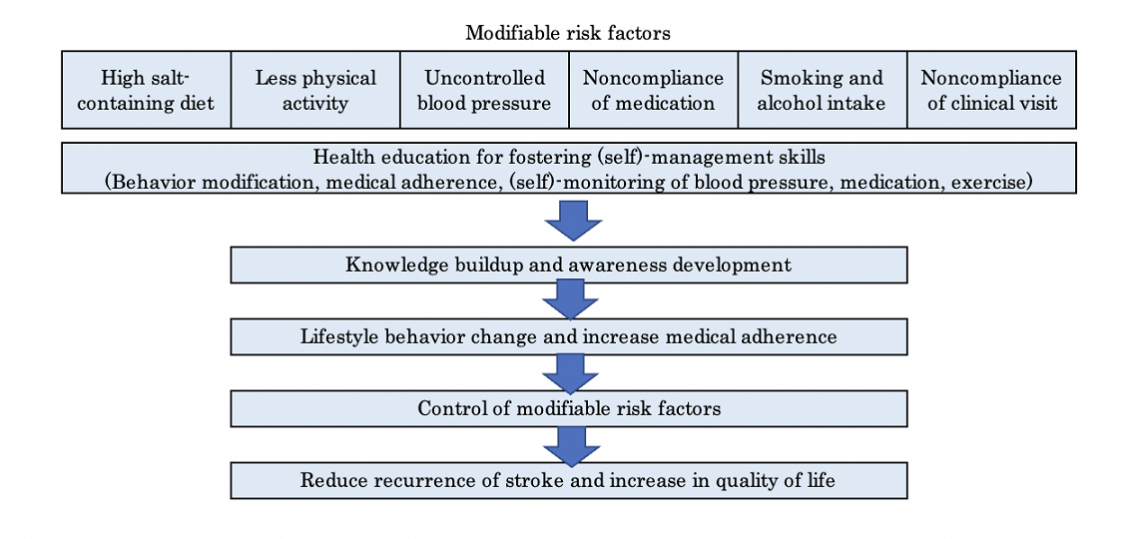
Conceptual framwork.

In Bangladesh, lack of patient and family education during and after hospital discharge has led to poor follow-up, including low compliance with clinical visits, medications, and lifestyle changes, resulting in negative outcomes [18]. From the nursing perspective, this study aims to reduce recurrence by fostering (self)-management skills through health education on modifiable risk factors of stroke. After patients or caregivers acquire knowledge and are aware the importance of health care management, their lifestyle behaviors can be changed. (Self)-monitoring of BP, medications, and clinical visits will enhance adherence to treatment and consequently control for the risk factors, increase QoL, and reduce stroke recurrence. Therefore, we hypothesized that health education of patients who had their first stroke and their family caregivers could reduce the stroke recurrence rate by controlling the modifiable risk factors compared to patients who did not receive health education. Health education during the hospital stay of patients who had a stroke and their continuous care after discharge are essential. Therefore, we developed a conceptual framework (Figure 1) to illustrate the factors that influence the recurrence of stroke, and we conducted postdischarge education for patients and families to prevent the recurrence of stroke for the first time in Bangladesh. The American Heart Association and Canadian guidelines recommended healthy lifestyle and management of vascular risk factors for the secondary prevention of stroke [19,20]. Meta-analyses of randomized controlled trials on intensive control of systolic BP [21,22] and improved antiplatelet therapy [23] reported significant reduction in the recurrence of stroke. Lifestyle-related behaviors such as diet, physical activity, and smoking contribute to vascular risk factors of stroke; however, there is knowledge gap on if modifying these behaviors significantly reduce stroke recurrence in stroke survivors [19]. Moreover, there is evidence that positive lifestyle change in intervention studies focused on improving recurrence prevention behaviors [17], but it is not established if lifestyle changes can reduce stroke recurrence. Patients’ medication adherence can be improved by providing information regarding the advantage of taking treatments, reminders, and (self)-monitoring [24]. Moreover, the (self)-management behavior of patients who had a stroke improved their self-efficacy, disease outcome, QoL, and self-satisfaction that could improve stroke recurrence [25]. It also increased patients’ self-confidence and well-being [26].

To enhance the knowledge and awareness of modifiable risk factors, we provided health education on various topics: (1) diet (reducing salt intake, maintaining a balanced diet, and managing weight) (Multimedia Appendix 1), (2) physical activity (increasing exercise or rehabilitation if patients are bedridden) (Multimedia Appendix 2), (3) smoking cessation and reducing alcohol consumption (Multimedia Appendix 3), (4) medication compliance and regular clinical visits (Multimedia Appendix 4), and (5) (self)-monitoring of BP (caregivers monitor BP if patients are unable to do so themselves) (Multimedia Appendix 5). Additionally, patients are educated about the importance of taking medication as prescribed and recognizing stroke symptoms. In Bangladesh, where the illiteracy rate is still high [27], the investigators developed an easy-to-understand study book named “Preventing Recurrence of Stroke: Friendly Health Education Booklet,” featuring illustrations and photos tailored to Bangladeshi culture and lifestyle. Based on the study book, videos were produced to ensure that even illiterate patients and caregivers could understand the content and watch them repeatedly. Furthermore, a 45-minute hands-on training session is provided for better comprehension. To ensure adherence to these (self)-management activities, the investigators provide patients with a digital BP machine, a salt-measuring spoon, and a medication box that divides the medicines for 1 week into morning, afternoon, evening, and bedtime doses. These tools are accompanied by a health education booklet and video, as well as a (self)-monitoring notebook for patients to utilize at home. The (self)-monitoring notebook is designed for patients or family caregivers to record daily monitoring data, including medication, BP readings, and any notable observations for a duration of 12 months. Nurses regularly contact patients to ensure they are measuring and recording data correctly, explaining how to interpret BP readings and their implications. The investigators recognize the critical importance of daily (self)-monitoring of BP to promote awareness of the patients’ condition and foster a sense of (self)-management. In Bangladesh, where the practice of measuring BP at home or even in hospitals is rare, investigators teach patients and caregivers how to measure BP, which is a new concept for the population who have never measured their BP levels before [28]. Another significant aspect is monitoring salt intake. The average daily salt consumption among Bangladeshi people ranges between 15 g/day and 21 g/day [29]. Due to the scarcity of processed and packaged foods in Bangladesh, where salt is the main seasoning, the concept of “measuring salt” is uncommon. Education on low-salt diets is virtually nonexistent in the country. Hence, it is crucial to teach individuals to comprehend and visualize “5 g of salt” by using a measuring spoon. Additionally, physical activities will be taught by a physical therapist.

## Methods

### Study Design

**Figure 2:**
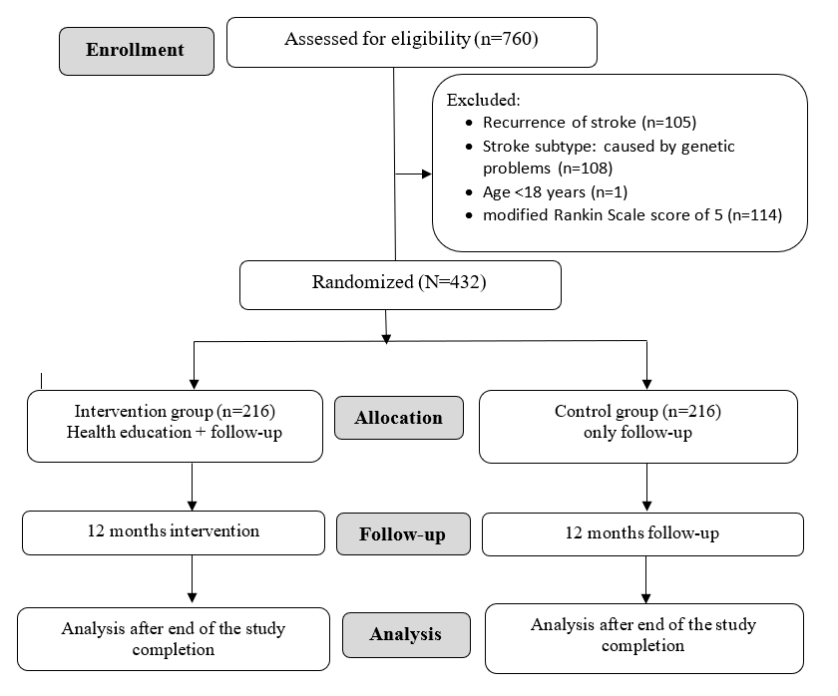
CONSORT (Consolidated Standards of Reporting Trials) chart.

This is an ongoing 12-month intervention study. This study follows a parallel (1:1), open-label, prospective, randomized controlled trial design, and adheres to the CONSORT (Consolidated Standards of Reporting Trials) guidelines [30]. The study flowchart is depicted in Figure 2. This study commenced in October 2022 and is expected to conclude in March 2024, with a total duration of 18 months. This trial is registered as ClinicalTrials.gov NCT05520034.

### Study Site, Population, and Sample

This study was conducted at the National Institute of Neurosciences & Hospital (NINS&H) in Dhaka, Bangladesh. NINS&H is a state-run tertiary care hospital in Bangladesh that treats patients with neurological disorders. The inclusion criteria for enrollment encompassed individuals who experienced their first stroke attack, regardless of whether it was the ischemic or hemorrhagic subtype. In Bangladesh, ischemic stroke accounts for over three-quarters of cases [4], and health education can be effective for patients with ischemic stroke classified as modified Rankin Scale (mRS) scores 0-2. For hemorrhagic stroke, considering the impact of self-management, patients with an mRS score below 3 were included. However, in the context of Bangladesh, family caregivers often provide care, and if family education is considered, patients with an mRS score of 4 were also included. Additionally, we included patients who have an mRS score of 0-4, were 18 years of age or older, and of any gender. Moreover, due to the limited availability of emergency hospital beds in Bangladesh, patients with an mRS score of 0-2, particularly those with lacunar infarcts, are often treated as outpatients. Hence, we included outpatient care in our study. In cases where patients were unable to care for themselves and relied on the support of family caregivers, consent was obtained from the caregivers. Primary caregivers, regardless of gender, who were 18 years of age or older, and resided with the patients were recruited. The exclusion criteria consisted of patients with a history of stroke recurrence, stroke caused by genetic factors or injury or accident, multiorgan failure, terminal illness, or previous participation in other clinical trials. Patients with an mRS score of 5, indicating severe disability requiring constant nursing care, were excluded [31]. In such cases, health education and follow-up may not have a significant impact. Additionally, individuals younger than 18 years, mentally unstable, cognitively impaired (diagnosed cases), and family caregivers who did not reside with their patients were also excluded from this study. When patients were admitted as inpatients or visited the outpatient department of the hospital, investigators and the research assistant (RA) nurses reviewed the participants’ admission or visiting files, including their current medications. Subsequently, the RA nurses visited the inpatient wards and brought the outpatients to a designated research room within the hospital.

### Patient Enrollment

A total of 760 patients were screened, and among them, 432 patients were enrolled. We randomly allocated 216 patients to the intervention group and 216 patients to the control group. After completion of 12 months follow-up, we will analyze the data.

### Randomization Schedule

A randomization schedule was used following the stratified randomization technique on a computer-generated series of numbers. On the basis of studies conducted in China [32,33], we stratified the patients into <65 years group and ≥65 years group, as the study site had no statistics about the age of the patients with recurrent stroke and types of strokes (hemorrhagic stroke, large artery atherosclerosis, cardioembolic, small vessel occlusion, lacunar, transient ischemic attack) because recurrence rates differed by the stroke type [6]. An experienced investigator who was not involved in this study generated the random allocation number by sequences. She distributed them in sealed opaque envelopes with serial numbers, which were kept in locked file cabinets in the hospital.

### Study Procedure

Enrollment occurred over a span of 6 consecutive days during morning hours, excluding government holidays, within a week. After determining eligibility and obtaining consent, the RA nurses opened the sealed randomization envelope in the presence of the participants. They collected demographic information, conducted physical examinations, and filled up questionnaires (Table 1).

**Table 1:** Study activities.

Schedule	Intervention group	Control group
At baseline	Data collection by RA^a^ nurses through structured questionnaires RA nurses provide knowledge-related health education before discharge or after the hospital visit: health education (face-to-face, 45 min) by RA nurse and exercise session by the physiotherapist RA nurses perform physical examination: BP^b^ level (systolic and diastolic) Blood tests: HbA_1c_^c^, total cholesterol, and HDL cholesterol (calculate non-HDL cholesterol)	Data collection by RA nurses through structured questionnaires Provide usual practice RA nurses perform physical examination: BP level (systolic and diastolic) Blood tests: HbA_1c_, total cholesterol, and HDL^d^-cholesterol (calculate non-HDL cholesterol)
Middle of study (6 months)	Data collection by RA nurses through structured questionnaires RA nurses provide refresher health education same as baseline RA nurses perform physical examination: BP level (systolic and diastolic) Blood tests: HbA_1c_, total cholesterol, and HDL cholesterol (calculate non-HDL cholesterol)	Data collection by RA nurses through structured questionnaires Provide usual practice RA nurses perform physical examination: BP level (systolic and diastolic) Blood tests: HbA_1c_, total cholesterol, and HDL cholesterol (calculate non-HDL cholesterol)
End of study (12 months)	Data will be collected by RA nurses through structured questionnaires RA nurses will perform physical examination: BP level (systolic and diastolic) Laboratory tests: HbA_1c_, total cholesterol, and HDL cholesterol (calculate non-HDL cholesterol)	Data will be collected by RA nurses through structured questionnaires RA nurses will provide health education at the end of the study, and a health education booklet will be given to them RA nurses will perform physical examination: BP value (systolic and diastolic) Laboratory tests: HbA_1c_, total cholesterol, and HDL cholesterol (calculate non-HDL cholesterol)

^a^RA: research assistant.

^b^BP: blood pressure.

^c^HbA_1c_: glycated hemoglobin.

^d^HDL: high-density lipoprotein.

### Intervention Group Study Plan

**Figure 3:**
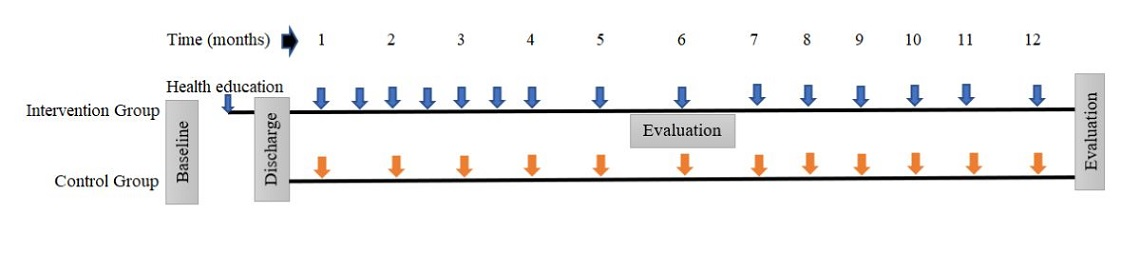
Follow-up and evaluation timeline (months) of the intervention and control groups.

After obtaining informed consent and baseline data, RA nurses provide health education through a booklet to patients and family caregivers and give them the booklet to follow the instructions at home. Subsequently, a video containing the risk factors of stroke and their management and prevention is shown to the patients and family caregivers. Health education sessions on booklet contents take around 20 minutes, and video show sessions take around 15 minutes. We provide these health education sessions 2 times after enrollment of the patients and 6 months after the follow-up visit in the hospital. Health education booklets guide patients on how to read food labels accurately for the prevention of stroke-related risk factors. Booklets have a reminder for the schedule date of the next clinic visits. There is also a notebook for recording daily health monitoring charts such as BP, exercise, and medication intake by the patients or caregivers. The video presentation includes simulation training on essential tasks such as how to measure BP and organize the medication boxes prescribed by physicians. We recommended placing the medications in our provided medication boxes to increase the adherence to doctors’ guidelines for consumption and using salt-measuring spoons. After that, an assigned physiotherapist provides about 15-minute exercise orientation sessions to show how to do physical exercises at home. A repeated session is conducted by the physicians and RA nurses at 6-month follow-ups for their memorization. RA nurses provide motivational SMS texts every month to the patients and caregivers to remind patients to measure their daily BP, reduce salt intake, avoid oily and fatty foods, consume more fruits and vegetables, exercise regularly, and adhere to their medication regimen. Additionally, RA nurses provide refresher health education twice a month in the first 3 months and then monthly in the next 9 months over a phone call to obtain information about the health condition of the patients and document in the questionnaires. Patients receive a reminder phone call on the fifth month to visit the hospital for the sixth month follow-up visit. At the 6-month follow-up visit (middle of study), if the patient and family caregiver are not able to come for any reason, RA nurses collect data and provide refresher health education over a phone call (Figure 3).

### Control Group Study Plan

The control group participants receive a monthly telephone call for 1 year from RA nurses solely to gather information about the patients’ health conditions and document the information in the questionnaires. At the fifth month, patients are reminded to visit the hospital for their sixth month follow-up visit (Figure 3). To mitigate the disadvantage faced by participants in the control group, after completing the study at the end point, they will be given the same health education booklet. If requested by the participants, a brief health education session will be provided based on the information contained in the booklet for their easy understanding.

### Outcomes and Data Collection Procedure

#### Primary Outcome

The primary end point is the recurrence rate of stroke at 12 months.

#### Secondary Outcomes

The secondary outcomes are (1) all adverse events (stroke and other events), (2) changes in the values of modifiable risk factors of BP, glycated hemoglobin, non–high-density lipoprotein cholesterol, and (3) changes in the scores of knowledge related to stroke in the investigator-developed questionnaire (this questionnaire consists of 10 items with “no/wrong answer,” “yes/correct answer,” and “don’t know” answers; the score ranges from 0 to 20, and a higher score indicates having better knowledge), (4) changes in scores in lifestyle behavior related to stroke in the investigator-developed questionnaire (this questionnaire consists of 14 items with a Likert scale to assess the frequency of behavioral changes; the score ranges from 0 to 37, and a higher score indicates having better lifestyle behavior), (5) changes in behavior in medication adherence by using Morisky Green Levine Medication Adherence Scale [34] (this questionnaire consists of 4 items with “yes=0” and “no=1” answers; the score ranges from 0 to 4, and a higher score indicates better compliance), and (6) changes in scores of QoL by using the QoL questionnaire (WHOQoL-BREF Bangla version [35]; each item scores from 1 [very poor or very dissatisfied] to 5 [very good or very satisfied] and ranges [mean of 2 items] from 1 to 5—a higher mean indicates a better QoL).

### Operational Definitions for This Study

#### Recurrence of Stroke

Recurrent stroke is defined as a new stroke event occurring at least 28 days after the stroke incident [32], which is confirmed by the physician who treats and diagnoses the patients in this study.

#### Adverse Events

All adverse events (all causes of death, cardiovascular events) refer to all cases that need to be diagnosed, for example, recurrence of stroke, new onset of acute myocardial infarction and unstable angina, heart failure with hospitalization, and peripheral arterial disease with hospitalization—those that are confirmed by the physician who treats and diagnoses the patients in this study.

#### Sample Size

Stroke recurrence rate in Bangladesh was 20% within 1 year in 2019 [8]. A study that evaluated the effectiveness of health education in preventing the recurrence of stroke showed 50% reduction among the intervention group compared to the control group [13]. The expected proportion of outcomes from the intervention group was assumed as P1=0.10; P2 (proportion of outcome from control group)=0.20; α=.05 (2-tailed), 1-β=.80; Z1-α/2=Z, α=1.96; and Z1-β=Z, β=.84. We found that the estimated sample size was 196 for each arm (a total 392 for both arms). By considering 10% dropout, the total sample size was 432 (216 for each arm).

### Study Preparation

#### Training of RA Nurses

Five RA nurses were recruited from NINS&H and another tertiary hospital, and 5 student nurses were recruited from 2 nursing schools for this study. Investigators provided protocol-specific theoretical and practical training to all RA nurses before the study initiation. They received training on physical measurements and were taught the motivational interviewing technique [36] to effectively encourage patients who had a stroke to adhere to instructions. We trained them how to educate patients to maintain daily activities and to send SMS text messages. Throughout the study, the RA nurses have been receiving monthly feedback sessions on the participants’ health education materials.

#### Data Monitoring and Quality Assurance

The investigators (medical doctors) are directly involved in patient management. The investigators and the RA nurses have regular biweekly meetings for study purpose. Pretesting of the questionnaires was done among the patients (5% of the sample of 432) who did not participate in the main study. Additionally, for regular quality control purpose, investigators independently check 5% of the study participants’ data on the same day by using field testing. The investigators check the process of taking consent, data collection, and physical examination by the RA nurses. The detected errors are corrected immediately at the field site. For any query, RA nurses consult with the investigators. RA nurses fill up the questionnaires and enter the data into a password-protected computer. Investigators check the data and resolve the queries.

### Ethics Approval

This study was approved by the institutional review board and the ethics review board of the NINS&H (IRB/NINSH/2022/151). All participants were explicitly informed about the study goals, significance, risks, and benefits, and their rights to participate in the study prior to the requirement. To participate in this study was entirely voluntary, and participants can withdraw at any point from their participation.

### Statistical Analysis

To compare the outcomes of the intervention group and the control group, we will conduct an intention-to-treat analysis and a full set analysis. A chi-square test will be performed for categorical data, and a 2-sided *t* test or Mann-Whitney *U* test will be employed for continuous variables, as appropriate, to compare the 2 groups. For the primary end point, the cumulative risk of recurrence and adverse events occurring in 12 months will be estimated using the Kaplan-Meier method and presented overall by age group and by stroke subtypes. For the secondary end points, an analysis of covariance will be conducted to assess the effect of the intervention on the outcomes by adjusting confounding variables. Changes in outcomes over time will be analyzed using repeated-measures analysis of variance as well as regression analysis. Friedman test will be performed to examine the changes within the groups. Associations between risk factors and outcomes will be tested using Cox proportional hazards models, adjusting for all baseline factors. Additionally, laboratory test and physical examination data will be analyzed using 2-sided *t* tests or 2-way repeated-measures analysis of variance, considering data normality. For the knowledge-related questionnaire, medication adherence–related questionnaire, and QoL, reliability checking will be performed, and total scores will be calculated. The significance level will be set at .05. Data analysis will be conducted using SPSS (version 26.0; IBM Corp).

## Results

The enrollment of the participants was completed in February 2023, and the study follow-up and data collection will continue until February 2024. A total of 432 participants agreed to participate in this study, with 216 allocated to the intervention group and 216 to the control group. Data cleaning and analysis are expected to be completed in May 2024.

## Discussion

### Overview

This study describes the protocol for a randomized controlled trial that aims to develop awareness, enhance knowledge, and make behavioral changes through face-to-face health education and phone calls among individuals who had their first stroke and were discharged from hospitals admitted from different regions of Bangladesh. To the best of our knowledge, this is the first research study conducted in Bangladesh to measure the effectiveness of health education and monthly follow-ups over mobile phone in reducing the recurrence of stroke. One of the most significant barriers to the effective treatment of stroke is the lack of awareness and education about modifiable risk factors, its complications, and the optimal way to treat stroke, which can be addressed by providing health education and monthly follow-ups over mobile phone for knowledge generation. Further, creating an awareness and employing behavior change communications among patients with stroke can reduce the recurrence of stroke and increase their QoL. Although stroke management is a significant burden on health practitioners in low-resource settings, RA nurses can be an important and underutilized resource for patient education. RA nurses have been shown to be effective in assisting people in improving their health habits. An RA nurse intervention among patients with modifiable risk factors such as hypertension and diabetes can increase patients’ awareness and help them control their BP and blood glucose levels. Monthly follow-ups as well as health education show a lot of potential in raising patient awareness and understanding of stroke disease as well as improving stroke knowledge.

### Strengths

We implemented various strategies to empower patients and caregivers, including training on BP measurement, medication box usage, salt intake measurement using spoons, and health education booklets. Self-monitoring of BP is facilitated by providing patients with a BP machine for daily measurements, which is generally not practiced in Bangladesh. Medication compliance is assessed using medication boxes, and accurate salt intake measurement will be facilitated by providing salt-measuring spoons. Most of the participants are not aware of the benefits of regular medication and low salt intake. Health education materials such as booklets and videos in Bengali are tailored to accommodate both literate and illiterate patients and their caregivers. Participants and even health care providers had no understanding to date about the advantage of health education in the health care system in Bangladesh. NINS&H is the only referral hospital in Bangladesh that patients attend from all over the country. Thus, our study findings can be generalized to all the patients who had a stroke in Bangladesh.

### Limitations

Our study has certain limitations. Although we provide education to the patients and their caregivers for their disease (self)-management at home, the level of education and understanding of each patient and his or her caregiver about the importance of monitoring risk factors would be different. This might influence the study outcomes. There will be heterogeneity at baseline in the level of illness, including level of consciousness, among patients with different subtypes of stroke. This could be a cause of outcome bias.

### Conclusions

Stroke recurrence can be caused by different modifiable risk factors. Thus, early detection and management of these risk factors are crucial for preventing subsequent stroke recurrences and for lowering morbidity and death. If our results show an enhancement in the study outcomes among patients with stroke (mRS score 0-4), health education can be integrated with monthly follow-ups as an effective tool for preventing stroke recurrence at the national level in Bangladesh. Further, we can improve patient knowledge and motivate patients with stroke regarding their health care practices to improve their QoL. If this health education program results in significant improvements in the QoL of patients who have had a stroke, then this program can be introduced and implemented in the health care system of Bangladesh.

## Appendix

Multimedia Appendix 1 Salt intake guidelines and balanced diet guides.

Multimedia Appendix 2 Physical activity education.

Multimedia Appendix 3 Smoking and alcohol intake cessation education.

Multimedia Appendix 4 Medication compliance.

Multimedia Appendix 5 Blood pressure monitoring guidelines.

## Abbreviations

BP: blood pressure
CONSORT: Consolidated Standards of Reporting Trials
mRS: modified Rankin Scale
NINS&H: National Institute of Neurosciences & Hospital
QoL: quality of life
RA: research assistant
