# Supplementary figures and images for: Effectiveness of a Health Education Program for Patients Who Had a Stroke and Their Caregivers by Controlling Modifiable Risk Factors to Reduce Stroke Recurrence in a Tertiary Hospital in Bangladesh: Protocol for a Randomized Controlled Trial
Source: JMIR Res Protoc. 2023 Dec 15;12:e51178. doi: 10.2196/51178 (PMC10757230; doi:10.2196/51178)

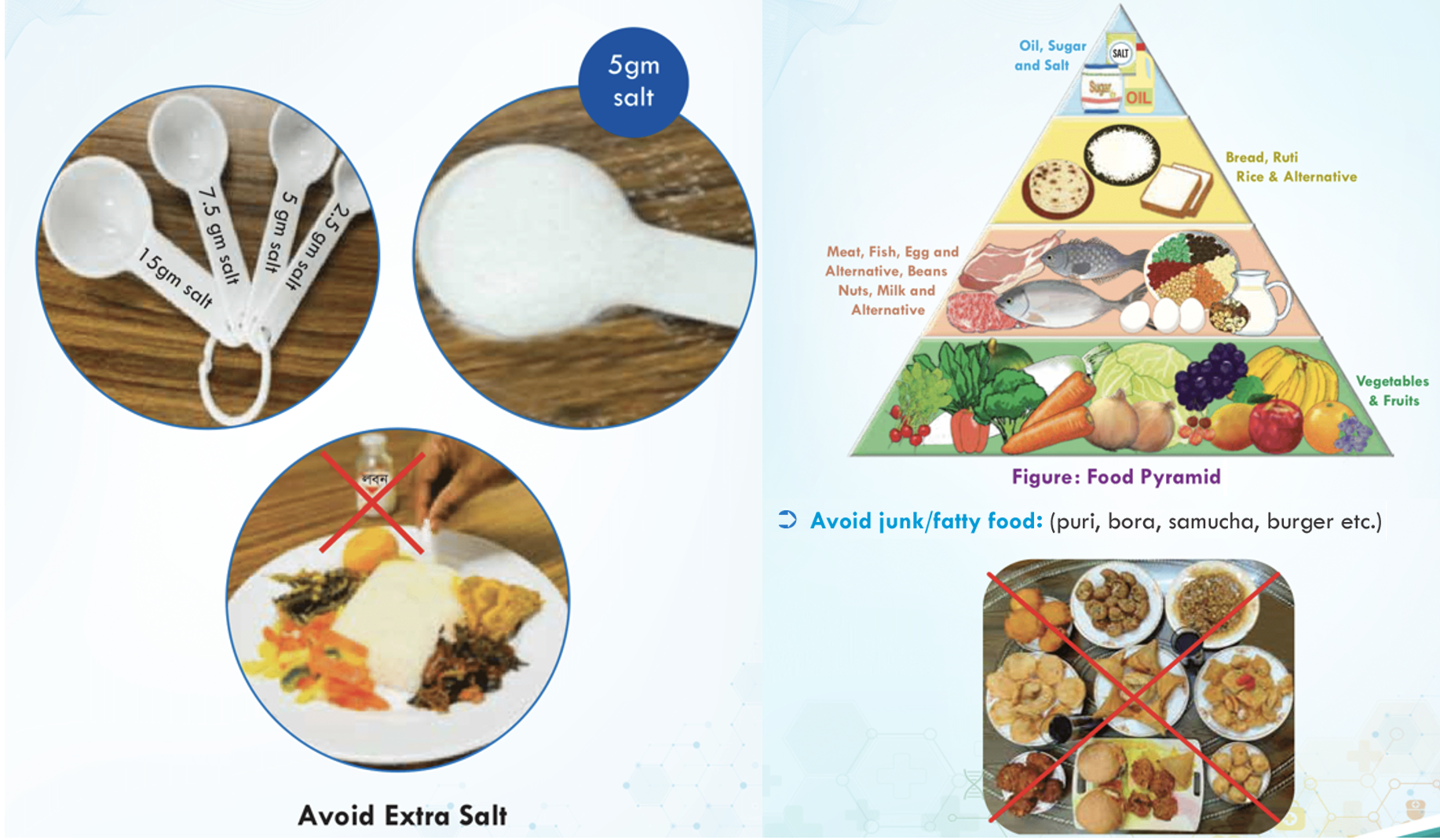

Supplement: Multimedia Appendix 1 [file resprot_v12i1e51178_app1.png]

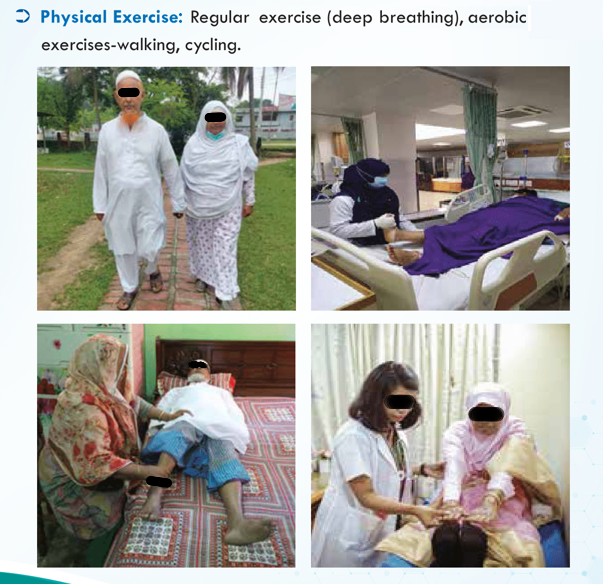

Supplement: Multimedia Appendix 2 [file resprot_v12i1e51178_app2.png]

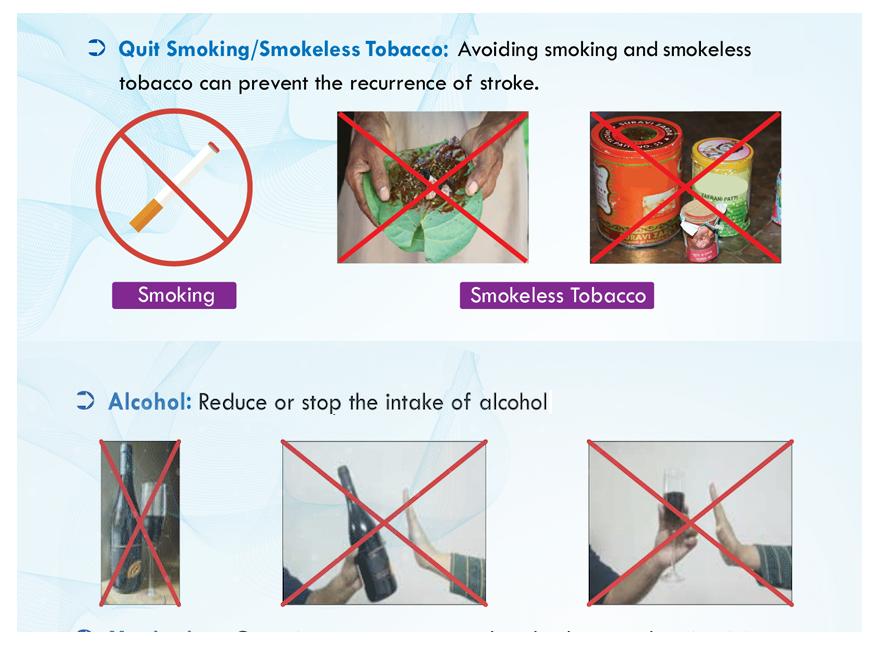

Supplement: Multimedia Appendix 3 [file resprot_v12i1e51178_app3.png]

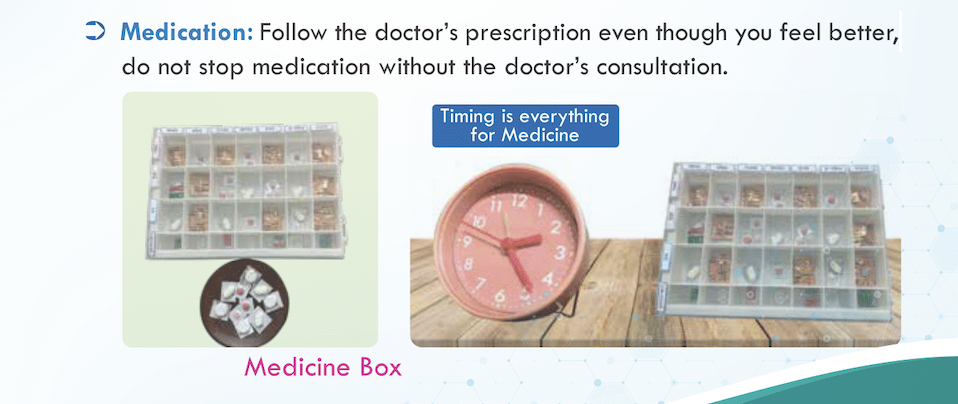

Supplement: Multimedia Appendix 4 [file resprot_v12i1e51178_app4.png]

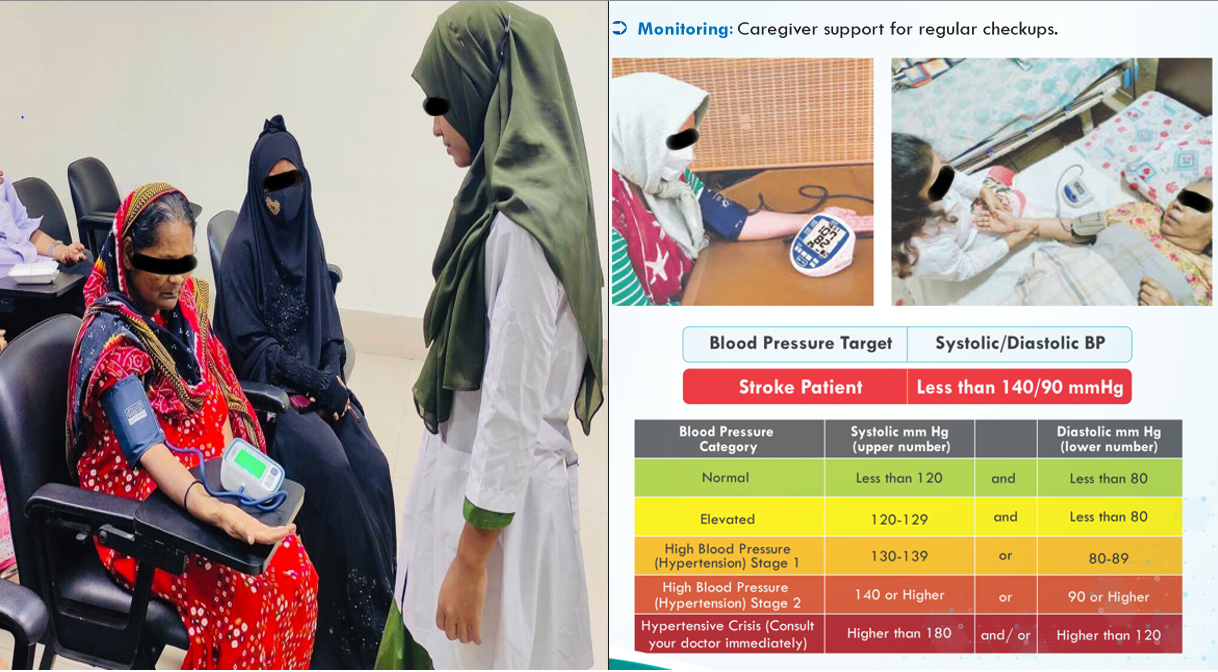

Supplement: Multimedia Appendix 5 [file resprot_v12i1e51178_app5.png]
